# Supplementary material for: Tnfaip2/exoc3‐driven lipid metabolism is essential for stem cell differentiation and organ homeostasis
Source: EMBO Rep. 2020 Dec 10;22(1):e49328. doi: 10.15252/embr.201949328 (PMC7788457; doi:10.15252/embr.201949328)
Supplement: Supplementary file 1 — Expanded View Figures PDF [file EMBR-22-e49328-s001.pdf]

Expanded View Figures

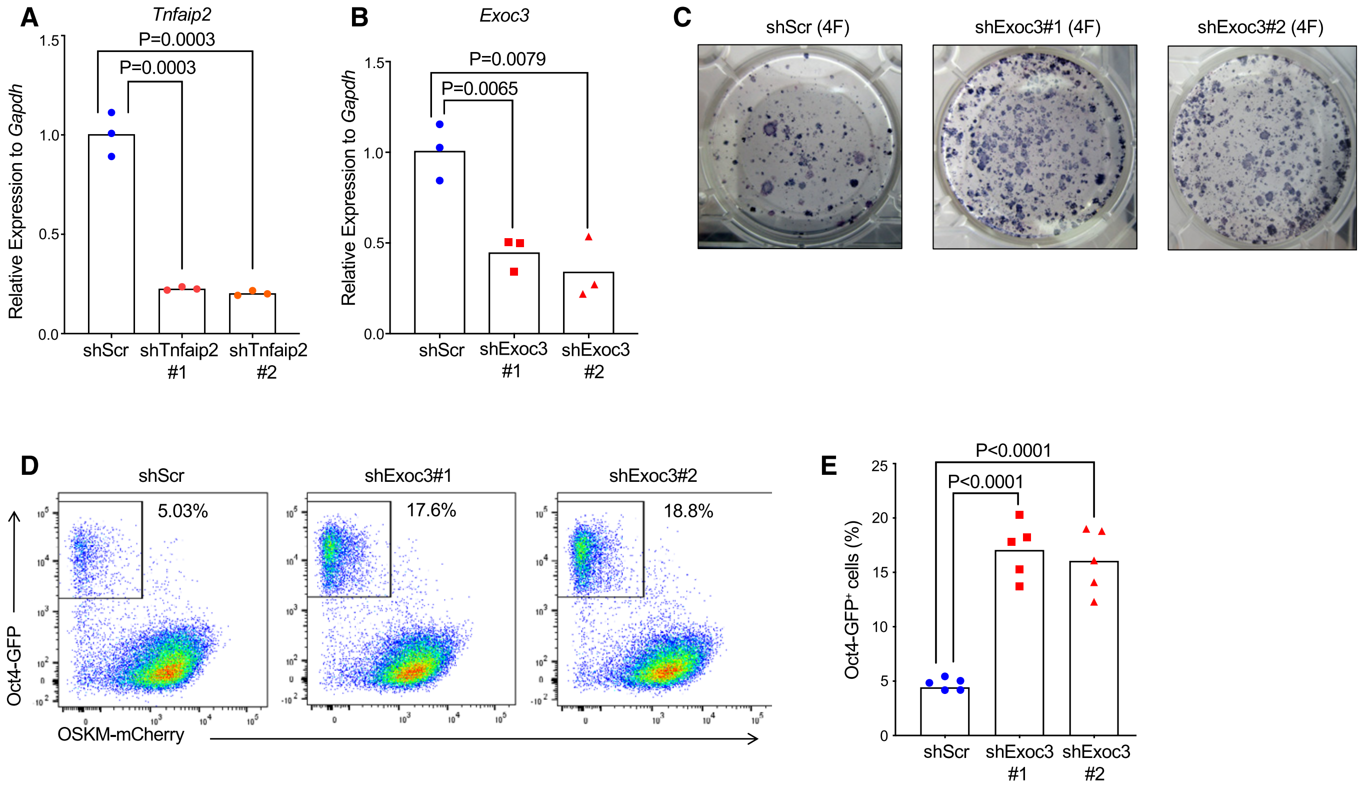

Figure EV1. Knockdown *Exoc3* enhances reprogramming of MEFs.

A RT–qPCR analysis on *Tnfaip2* expression in MEFs that were infected with shRNAs against *Tnfaip2*. The shTnfaip2#1 was from the screening library (Fig 1), and the shTnfaip2#2 was independently designed. Expression levels were determined in FACS-purified, infected cells (BFP-positive) on day 4 after infection [n = 3 biological replicates; log-transformed data were normally distributed ($P > 0.05$ as per Shapiro–Wilk test) and analyzed by one-sided t-test with Holm–Sidak correction for multiple testing].

B RT–qPCR analysis on *Exoc3* expression in MEFs that were infected with two different shRNAs against *Exoc3*. Expression levels were determined in FACS-purified, infected cells (BFP-positive) on day 4 after infection [n = 3 biological replicates; log-transformed data were normally distributed ($P > 0.05$ as per Shapiro–Wilk test) and analyzed by one-sided t-test with Holm–Sidak correction for multiple testing].

C–E *Oct4*-eGFP reporter MEFs were infected with shRNAs against *Exoc3* or a control (scrambled) shRNA and co-infected with a polycistronic vector expressing the four reprogramming factors (OSKM). Double-infected cells were FACS-purified and were allowed to reprogram for 14 days in total and were then analyzed: (C) Representative images of AP staining of iPS colonies (n = 3–4 biological replicates), (D) representative FACS profiles, and (E) histogram on the percentage of *Oct4*-GFP$^+$ iPSCs in MEF cultures that were infected with shRNAs targeting *Exoc3* or a scrambled shRNA control [n = 5 biological replicates; data were normally distributed ($P > 0.05$ as per Shapiro–Wilk test) and analyzed by one-sided t-test with Holm–Sidak correction for multiple testing].

© 2021 The Authors

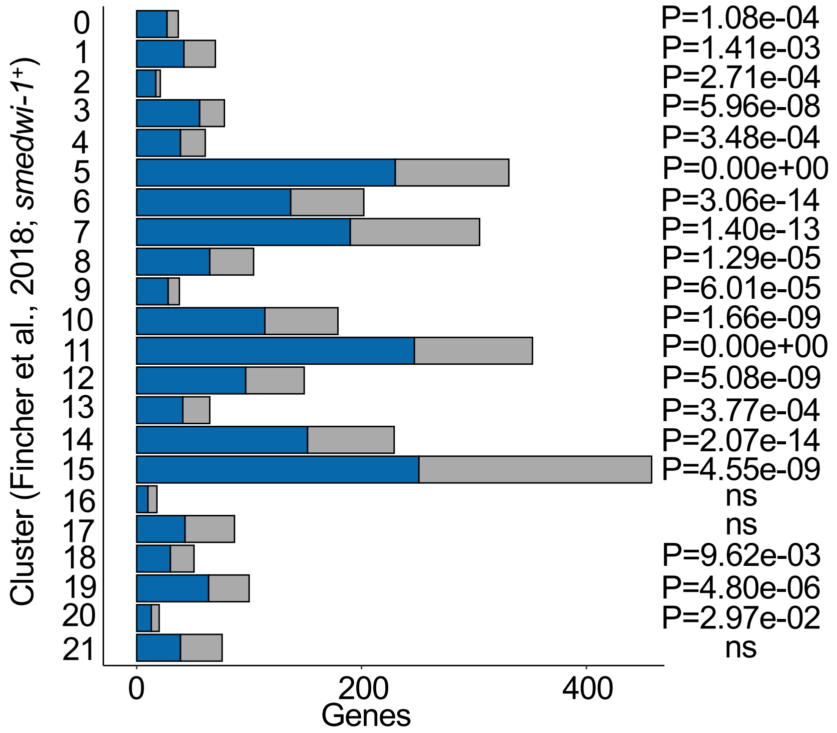

Figure EV2. *Smed-exoc3* depletion alters gene expression in planarian neoblast.

RNA-seq was conducted on freshly isolated X1 cells from *exoc3(RNAi)*-treated and X1 fraction of *gfp (RNAi)*-treated planarians on day 38 of the injection protocol. The transcriptome analysis results were compared with previously published gene expression profile. The bar-graph depicts the overlap (blue) of DEGs in X1 cells of *Smed-exoc3*-depleted planarians versus X1 cells of controls ($n = 3$ biological replicates; adjusted $P < 0.05$) with previously published gene expression profiles in progenitor cells that exhibit expression of the neoblast marker *smedwi-1* (referred to as *smedwi-1*$^{+}$) but a distinct gene expression signature from the neoblast population indicating that these cells represent a population of progenitor cells differentiating into different lineages (Fincher *et al*, 2018). The sum of overlapping (blue) and set-exclusive (gray) genes represents the total number of genes of a cluster that were analyzed in this study. *P*-values right of the bars represent significance of overlap (hypergeometric test).

 © 2021 The Authors

A

Reference full length amino acid sequences for mouse *Tnfaip2*

>*Tnfaip2* (WT)

MLKMVTFFQGFPGQQSVPGTLNFAVSPQKPRSTSEAESETSMSEASSEDLMPSPEAPDGEEESAKKKEK
KSKGLANMFSVFTKGKKKKKDQPRLSDLEVQPKPRPELDGPLPTVEELKEALEHGRLEVAWQVLALERQL
EAAAAAGGMSNEELVWRQSKVEALYVLLCDQVLGVLRRPLEAAPERLSQALAVVSQEELEDRRASGGPLA
AALEATRPRRWLQRWRGVVAEVAAERLDAQPATAPEGRSEAESRFLHMGRTMKEDLEVVVERLKPLFPDE
FNVVRTYAESYHYHFASHLCALAQFELCERDTYLLLLWVQNLYPNDILNSPKLAQELQGVGLGSLLPPKQIR
LLEAMFLSNEVTSVKQLMARALELESQRWTQDVAPQSLDGHCHSELAIDILQIISQGQTKAENITSDVGMQI
KQLLLVELAALLRSYQRAFDEFLEKSKLLRNYRVNIMANINNCLFFWTSVEQKWQISHDSLNRLLEPLKDLK
AHGFDTLLQSLFLDLKPLFKKFTQTRWANPVETLEEIITTVSSSLPEFSELQDCFREELMETVHLHLVKEYIIR
LCKRRLVLKTAEQQQQLARHILANADAIQGFCTENGSTATWLHRALPMIAEIIRLQDSSAIKIEVATYATWYPD
FSKGHLNAILAIKGNLPSSEVRSIRNILDINTGVQEPPRPLFSLIKVT

Altered amino acid sequences for mouse *Tnfaip2* generated by CRISPR

>*Tnfaip2*$^{-/-}$

MLKMVTFFQGFPGQQSVPGTLNFAVSPQKPRSTSEAESETSMSEASSEDLMPSPEAPDGEEESAKKKEK
KSKGLANMFSVFTQREKEKKGPAQIIRSGSAAQAQARVRWSTAHR*AVAAAGAGTEGRGWSRFGQCR

QREKEK: Altered reading frame start site

*: Early stop codon

B

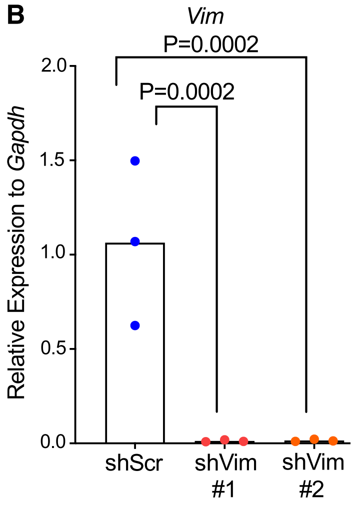

Figure EV3. ***Tnfaip2*$^{-/-}$ ES cells exhibit downregulation of *Vim* during *in vitro* differentiation.**

A CRISPR/Cas9-mediated genome editing to derive *Tnfaip2* knockout (*Tnfaip2*$^{-/-}$) ES cells. The full-length amino acid sequence of wild-type (WT) *Tnfaip2* is shown (upper panel). The CRISPR/Cas9-mediated targeting of the 2nd Exon of *Tnfaip2* with frameshift mutation (highlighted in yellow) and insertion of stop codon (asterisk in green) to generate *Tnfaip2*$^{-/-}$ ES cells (lower panel).

B RT–qPCR analysis on *Vim* expression in MEFs that were infected with two independent shRNAs against *Vim*. Expression levels were determined in FACS-purified, infected cells (BFP-positive) on day 4 after infection [n = 3 biological replicates; log-transformed data were normally distributed ($P > 0.05$ as per Shapiro–Wilk test) and analyzed by one-sided t-test with Holm–Sidak correction for multiple testing].

© 2021 The Authors
